# Trends of Thyroid Cancer in Israel: 1980–2012

**DOI:** 10.5041/RMMJ.10228

**Published:** 2016-01-28

**Authors:** Lital Keinan-Boker, Barbara G. Silverman

**Affiliations:** 1 Israel National Cancer Registry, Israel Center for Disease Control, Ministry of Health, Ramat Gan, Israel;; 2 School of Public Health, Faculty of Social Welfare and Health Sciences, University of Haifa, Haifa, Israel;; 3 School of Public Health, Sackler Faculty of Medicine, Tel Aviv University, Tel Aviv, Israel

**Keywords:** Israel, papillary carcinoma, thyroid cancer incidence, thyroid cancer mortality, thyroid cancer survival

## Abstract

**Objectives:**

Thyroid cancer incidence is increasing worldwide, while mortality from thyroid cancer is stable or decreasing. Consequently, survival rates are rising. We describe time trends in the incidence, mortality, and 5-year survival of thyroid cancer in Israel in 1980–2012, in light of the global trends.

**Methods:**

Israel National Cancer Registry database provided information regarding thyroid cancer incidence and vital status, which enabled computation of survival rates. The Central Bureau of Statistics database provided information on thyroid cancer mortality. Incidence and mortality rates were age-adjusted and presented by population group (Jews/Arabs) and gender. Relative 5-year survival rates which account for the general population survival in the corresponding time period were presented by population group and gender. Joinpoint analyses were used to assess incidence trends over time.

**Results:**

In 1980–2012 significant increases in the incidence of thyroid cancer were observed, with an annual percent change (APC) range of 3.98–6.93, driven almost entirely by papillary carcinoma (APCs 5.75–8.86), while rates of other types of thyroid cancer remained stable or decreased. Furthermore, higher rates of early detection were noted. In 1980–2012, a modest reduction in thyroid cancer mortality was observed in Jewish women (APC −1.07) with no substantial change in Jewish men. The 5-year relative survival after thyroid cancer diagnosis has increased to ≥90% in both population groups and both genders.

**Conclusions:**

The Israeli secular trends of thyroid cancer incidence (increasing), mortality (mostly stable), and survival (modestly increasing) closely follow reported global trends.

## INTRODUCTION

Thyroid cancer affects cells within the thyroid gland and is usually categorized into well-differentiated tumors, i.e. papillary carcinoma (accounting for approximately 75%–85% of all cases) and follicular carcinoma (10%–20% of all cases), and less differentiated tumors, i.e. medullary carcinoma (3%–10% of all cases) and anaplastic carcinoma (1%–3% of all cases).^1^ Prognosis for thyroid cancer varies by histologic diagnosis. Ten-year survival for papillary carcinoma is approximately 98%, and for follicular carcinoma 92%.However, the 10-year survival rates for other types of thyroid cancer are considerably lower: 80% for medullary carcinoma and 13% for anaplastic tumors. While tumors of other histological origins, such as lymphoma and sarcoma, may also present in the thyroid gland,^2^ they are not generally included in calculations of thyroid cancer incidence and survival.

The etiology of thyroid cancer is largely unknown, but some risk factors for the disease have been identified. Exposure to ionizing radiation (e.g. in medical procedures, including radiotherapy given in order to treat a previous cancer, fallout from power plant accidents or nuclear weapons, exposure in occupational settings) is an established risk factor for thyroid cancer.^3^ Other risk factors include gender (thyroid cancer occurs about three times more often in women compared to men), age (the incidence peaks at ages 60–70 in men and 40–50 in women), benign and autoimmune thyroid diseases, genetic factors, and a family history of thyroid cancer or of other cancers.^4^ Other potential risk factors are taller height and higher BMI (especially in women),^5^–^7^ certain dietary exposures,^5^–^7^ and reproductive factors.^7^,^8^

In 2012, a total of 298,102 thyroid cancer patients were diagnosed throughout the world (age-standardized incidence rate of 4.0/100,000), and a total of 39,771 subjects died of the disease (age-standardized mortality rate of 0.5/100,000).^9^ Based on Globocan 2012 data, Israeli men ranked eighth (age-standardized rate, ASR, 5.4/100,000) and Israeli women, tenth (ASR, 17.2/100,000) in the world in 2012 with respect to thyroid cancer incidence. As for thyroid cancer mortality, Israeli men ranked ninth (ASR, 0.4/100,000), together with Korea, Austria, Belarus, and Costa Rica, and Israeli women ranked sixth (ASR, 0.7/100,000) in the world in 2012.^9^ A recent report on thyroid cancer incidence and mortality in the world, based on the dataset of the World Health Organization, identifies Israel as one of the countries with the highest incidence and mortality rates of the disease for both males and females.^10^ Indeed, in Israel the incidence of thyroid cancer is relatively high; in 2012, currently the last year with complete cancer incidence data in the Israel National Cancer Registry (INCR), thyroid cancer was among the most common cancers in women and ranked fifth (5% of all invasive tumors) in Jewish women and third (8% of all invasive tumors) in Arab women. However, although common (especially in women), as a cause of mortality thyroid cancer in Israel is rather rare; deaths due to thyroid cancer in 2012 accounted for approximately 0.5% of all cancer deaths.^11^ Nevertheless, as mentioned earlier, Israeli mortality rates for thyroid cancer are relatively high compared to other countries.^10^

In most countries, a steady increase in the incidence of thyroid cancer has been observed during the last decades in both men and women. The increase is mainly due to an increase in the incidence of papillary carcinoma. Mortality rates have reportedly dropped or remained stable,^10^ and as a consequence survival rates for thyroid cancer are increasing.

The main objective of the current study is to describe time trends in the thyroid cancer incidence, mortality, and survival in Israel in the three decades 1980–2012, and to discuss the findings in light of global trends.

## METHODS

The Israel National Cancer Registry (INCR) was established in 1960, and since 1982 reporting of all newly diagnosed cancers in Israeli residents to the registry has been mandatory by law. The data collected by the INCR are derived from multiple sources and include demographic information (gender, date of birth, country of birth, dates of immigration to Israel and of death if applicable), date and location of cancer diagnosis, anatomical site and histological type of the malignant tumor, disease stage at diagnosis, and therapy at diagnosis. The completeness of the INCR is estimated to be approximately 94% for solid tumors.^12^

Because the INCR database is linked annually with that of the population registry to ascertain the vital status of the registered subjects, survival rates can be computed. In order to take into account the survival rates of the general population, relative survival rates are computed by dividing the observed survival of cancer patients by the expected survival in the general population adjusted for age, sex, and ethnicity, based on national mortality data.

Stage at diagnosis is recorded in the registry according to US Surveillance, Epidemiology and End-Results (SEER) project Summary Staging,^13^ which defines tumors as *in-situ*, locally spread, regionally spread, and distantly spread (metastatic).

The Central Bureau of Statistics (CBS) collects information on mortality and codes the specific causes of death. Data on thyroid cancer mortality rates were obtained for 1980 through 2012. In addition, the CBS provides annual population demographic data, which enable the calculation of rates in general and rates by specific age and population groups.

We searched the INCR database for all cases of thyroid cancer (ICD-O-3 site code 73.9) diagnosed during the period 1980–2012. We grouped thyroid cancers into histologic groups as follows: (1) Papillary carcinoma (ICD-O-3 8050, 8260, 8340–8341, 8343–8344, 8350); (2) Follicular carcinoma (ICD-O-3 8290, 8330–8332, 8335); (3) Medullary carcinoma (ICD-O-3 8345–8346, 8510); and (4) Anaplastic carcinoma (ICD-O-3 8012, 8020–9021, 8030–8032).

We also searched the CBS database for all thyroid cancer deaths in the years 1980–2012, based on ICD-9 code 193 (up to 1997) and on ICD-10 code C73 (1998 onwards).

We compared the two ethnic groups (Jews and Arabs) with respect to thyroid cancer incidence and mortality, age-standardized by the world standard population (“Segi”).^14^

Joinpoint analysis^15^ was used to identify significant changes in thyroid cancer trends and calculate annual percent change in age-standardized rates for the period 1980–2012, setting a minimum of one and a maximum of three joinpoints. The log transformation of the age-standardized incidence rate was used as the dependent variable, stratified by gender and population group. Because log-transformation cannot be performed in cases of ASR=0, in situations in which no cases were observed for a particular population group in a given year, we replaced the zero ASR with the value 0.000001. Population groups for which the average number of cases per year was less than 20 were excluded from joinpoint analysis.

Relative survival rates were computed for thyroid cancer cases as the ratio of 5-year survival in cancer patients to the survival expected in the general population, matched by population group, gender, age group, and time period, based on national mortality rates published by the CBS.

The alpha level was set as 0.05 for all analyses, and all tests were two-sided. All analyses were done using the SAS Server Microsoft Windows NT Version 5.2.

## RESULTS

### Thyroid Cancer Incidence

We identified a total of 17,055 cases of thyroid cancer diagnosed in Israeli residents during the study period, 1980–2012. The number of cases per year and age-standardized rates are presented in Table 1. Throughout the years, the age-standardized incidence of thyroid cancer rose in all population groups, most markedly among women (from 7.28/100,000 in 1980 to 18.05/100,000 in 2012 in Jewish women and from 2.50/100,000 in 1980 to 11.50/100,000 in 2012 in Arab women).

**Table 1.** Numbers and Age-adjusted Incidence Rates (per 10^5^) of Thyroid Cancer in Israel by Population Group and Gender: 1980–2012.^*^

**Year**	**Jewish Subgroup**				**Arab Subgroup**			
	**Male**		**Female**		**Male**		**Female**	
	**Count**	**ASR^†^**	**Count**	**ASR^†^**	**Count**	**ASR^†^**	**Count**	**ASR^†^**
1980	38	2.28	125	7.28	<5	2.25	5	2.50
1981	42	2.60	103	5.86	0	–	9	4.12
1982	49	2.95	116	6.33	<5	1.51	<5	1.43
1983	43	2.54	87	4.95	<5	0.89	5	1.69
1984	41	2.24	123	6.79	<5	1.02	5	2.20
1985	43	2.51	133	7.24	0	–	11	4.24
1986	50	2.75	110	5.84	2	0.75	<5	3.25
1987	35	1.79	134	7.18	<5	0.25	2	0.72
1988	35	1.80	126	6.57	<5	0.61	10	3.08
1989	53	2.85	144	7.51	5	1.36	9	3.47
1990	74	4.20	197	9.67	<5	1.02	14	4.73
1991	63	3.12	185	8.45	<5	1.06	13	3.72
1992	59	2.75	202	8.77	<5	1.34	22	6.99
1993	78	3.55	203	8.57	5	1.41	14	3.36
1994	72	3.15	242	9.91	10	2.98	16	3.76
1995	82	3.54	252	10.1	6	1.9	21	4.73
1996	96	3.82	270	10.67	6	1.33	30	6.30
1997	94	3.78	271	10.12	5	1.32	34	6.87
1998	88	3.35	317	11.98	<5	0.57	40	8.25
1999	90	3.35	307	10.35	12	3.57	20	4.61
2000	89	3.33	357	12.34	14	3.35	42	9.61
2001	102	3.77	374	12.93	14	3.34	34	6.88
2002	115	4.15	378	12.50	12	2.33	48	10.30
2003	121	4.27	377	12.78	15	3.00	42	7.98
2004	159	5.65	449	14.81	11	2.16	43	7.75
2005	144	4.96	437	14.11	18	4.16	50	9.46
2006	131	4.38	481	15.62	22	5.21	59	10.57
2007	146	4.8	542	17.02	17	3.26	60	10.22
2008	184	6.07	571	17.81	17	3.57	74	11.60
2009	186	5.84	581	17.95	20	3.49	96	15.25
2010	203	6.47	596	17.71	28	4.27	72	11.14
2011	210	6.47	605	17.77	20	3.24	77	11.30
2012	209	6.19	624	18.05	26	3.62	84	11.50

* Accurate count not presented if cases number per year is lower than 5.

† ASR, age-adjusted rates (according to the world standard population “Segi”) per 100,000.

**Figure 1.**
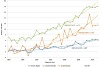
**Joinpoint Analysis of Time Trends in the Age-standardized Incidence of Thyroid Cancer by Population Group^†^ and Gender: Israel, 1980–2012.** ^*^ Statistically significant at the α<0.05 level. ^†^ The low annual number in Arab men precluded trend analysis. However, the secular trend closely followed that of the other population groups. APC, annual percent change.

Joinpoint analysis demonstrated significant increases in thyroid cancer overall for the study period among Jewish men, Jewish women, and Arab women (annual percent change (APC) in incidence, 3.98, 4.21, and 6.93, respectively) (Figure 1). Among Arab men, the small number of cases per year (less than 20 in most years) precluded trend analysis.

**Figure 2.**
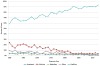
**Distribution of Thyroid Cancer Cases, by Histologic Type (% of All Cases): Israel, 1980–2012.**

**Figure 3.**
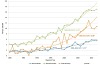
**Joinpoint Analysis of Time Trends in the Age-standardized Incidence of Papillary Thyroid Cancer by Population Group^†^ and Gender: Israel, 1980–2012.** ^*^ Statistically significant at α<0.05 level. ^†^ The low annual number in Arab men precluded trend analysis. However, the secular trend closely followed that of the other population groups. APC, annual percent change.

**Figure 4.**
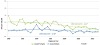
**Joinpoint Analysis of Time Trends in the Age-standardized Incidence of Non-Papillary Thyroid Cancer by Gender^†^: Israel, 1980–2012.** ^*^ Statistically significant at the α<0.05 level. ^†^ The low annual number in the Arab population precluded trend analysis. APC, annual percent change.

When thyroid cancer histological types are analyzed, it is obvious that the increase in incidence is not uniform across all types. In the total study period, 1980–2012, 80% of all cases were classified as papillary carcinoma. However, the proportion of cases classified as papillary carcinoma rose steadily, from approximately 60% in 1980 to 93% in 2012, while the proportions of other histological types of thyroid cancer decreased or remained stable (Figure 2). The increased proportion of papillary carcinoma also translated into increased incidence rates, a trend that was apparent in both population groups and in both genders (Figure 3). Joinpoint analysis clearly shows that the increases in the incidence of papillary thyroid cancer are pronounced in Jewish men, Jewish women, and Arab women (APCs of 5.75, 5.51, and 8.86, respectively) (Figure 3). Among Arab men, the small number of cases per year precluded trend analysis. For non-papillary thyroid cancers (i.e. follicular, medullary, and anaplastic, combined), significant decreases in incidence occurred in the Jewish male and female population (Figure 4) with APCs of −2.58 and −3.44, respectively. In the Arab population, the small number of non-papillary thyroid cancers reported annually precluded trend analysis.

Thyroid cancer is mostly diagnosed in middle-aged subjects. In 2012, the age-specific incidence of thyroid cancer in Jewish men was highest for the 50–74 age group (*n*=209), with a range of 17.34–25.13/100,000. In Jewish women, the age-specific incidence of thyroid cancer was highest for the 40– 69 age group (*n*=624), with a range of 35.49–54.10/100,000. The mean age at diagnosis of thyroid cancer for the period 2005–12 was 54.0 for Jewish men and 51.1 for Jewish women. For the Arab–Israeli population, in 2012 the age-specific incidence of thyroid cancer in men was highest in the 40–54 age group (*n*=26), with a range of 12.17–12.78/100,000; in women, the highest age-specific rates were observed for the 35–59 age group (*n*=84), with a range of 22.01–32.26/100,000. The mean age at diagnosis for the period 2005–12 was 46.4 for Arab men and 41.1 for Arab women. The age difference at diagnosis between the two population groups was significant for both genders (*P*<0.0001).

Papillary cancer, the most commonly diagnosed type of thyroid cancer, tends also to be diagnosed in middle age. The mean age at diagnosis of papillary thyroid cancer during the period 2005–12 was 52.9 for Jewish men and 50.0 for Jewish women, 44.8 for Arab men and 41.0 for Arab women. As was the case with thyroid cancer overall, Jewish men and women diagnosed with papillary thyroid carcinoma were significantly older at the time of diagnosis than their Arab counterparts (*P*<0.001).

From 2000 to 2012, we noted a progressive improvement in the proportion of thyroid cancer cases for which SEER summary stage could be determined. In 2000, there was insufficient information for the determination of stage in 56% of cases; by 2012, this proportion had dropped to 21%. Of cases for which stage could be determined, the proportion with local involvement (i.e. early-stage tumors) doubled, rising from 28.8% in 2000 to 56.1% in 2012, while the proportion with distant spread (i.e. metastatic tumors) considerably dropped from 10.9% in 2000 to 1.7% in 2012.

Trends in stage at diagnosis of papillary thyroid cancer mirrored those of thyroid cancer overall. Of cases for which stage could be determined, the proportion with local involvement rose from 29.9% in 2000 to 56.8% in 2012, while the proportion with distant spread dropped from 5.9% in 2000 to 1.6% in 2012.

### Thyroid Cancer Mortality

**Figure 5.**
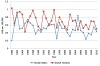
Age-standardized Mortality Due to Thyroid Cancer in the Jewish-Israeli Population by Gender*: Israel, 1980–2012. ^*^ The small numbers in the Arab subgroup produced unstable rates and thus are not shown.

We identified a total of 1,454 deaths attributed to thyroid cancer during the study period, 1980–2012. The number of cases per year and age-standardized rates are presented in Table 2 and Figure 5, respectively. Over the period of the study, the age-standardized mortality attributed to thyroid cancer in the Jewish population varied widely in Jewish men. Joinpoint analysis indicated an annual percent change (APC) in adjusted mortality of −0.84 (NS). Among Jewish women a steady decline in adjusted mortality occurred during the study period (from 1.03/100,000 in 1980 to 0.50/100,000 in 2012, APC −1.07, *P*<0.05). Mortality rates in the Arab population subgroup, among whom the annual number of deaths was in the range 0–3 in males and 0–5 in females, are unstable.

**Table 2.** Number and Age-adjusted Mortality Rates (per 10^5^) of Thyroid Cancer in Israeli Jews^*^ by Gender: 1980–2012.^†^

**Year**	**Male**		**Female**	
	**Count**	**ASR^‡^**	**Count**	**ASR^‡^**
1980	<5	0.20	24	1.03
1981	11	0.60	15	0.64
1982	19	0.88	20	0.92
1983	11	0.50	23	0.94
1984	10	0.52	21	0.87
1985	13	0.64	10	0.43
1986	14	0.74	16	0.68
1987	12	0.65	22	0.83
1988	12	0.59	27	0.97
1989	9	0.37	21	0.79
1990	11	0.48	16	0.56
1991	10	0.42	14	0.48
1992	14	0.53	28	0.95
1993	12	0.50	25	0.77
1994	17	0.63	24	0.59
1995	16	0.61	29	0.71
1996	18	0.62	42	0.99
1997	8	0.25	21	0.53
1998	21	0.66	33	0.80
1999	16	0.51	25	0.68
2000	16	0.52	25	0.56
2001	12	0.40	26	0.48
2002	11	0.31	38	0.87
2003	17	0.49	32	0.70
2004	19	0.46	34	0.72
2005	28	0.84	28	0.48
2006	17	0.46	43	0.70
2007	16	0.41	29	0.52
2008	11	0.24	32	0.61
2009	14	0.39	25	0.49
2010	16	0.35	39	0.70
2011	24	0.55	42	0.69
2012	20	0.44	33	0.50

* In the Arab subpopulation (both males and females) annual counts were lower than 5, and therefore the data are not presented.

† Accurate count not presented if case number per year is lower than 5.

‡ ASR, age-adjusted rates (according to the world standard population “Segi”) per 100,000.

The mean age of death due to thyroid cancer during the period 2005–12 (*n*=342) in Jewish men and women was 71.3 and 76.1, respectively. In Arab men and women the mean age at death from thyroid cancer was 63.8 and 64.4, respectively. The differences in the mean age at death between Jews and Arabs were not statistically significant for men (*P*=0.198) but did reach statistical significance for women (*P*=0.0001).

### Thyroid Cancer Relative Survival

The 5-year relative survival from thyroid cancer is high and has increased with time. For Jewish men diagnosed in the years 1991–5, 1996–2000, and 2001–6, the 5-year relative survival rates were 84.3%, 85.2%, and 90.3%, respectively. For Jewish women the corresponding figures were 90.6%, 92.9%, and 95.0%, respectively.

For Arab men diagnosed in the years 2001–6, the 5-year relative survival rate was 96.2% (survival rates for the years 1991–5 and 1996–2000 were not computed due to small numbers). In Arab women, 5-year relative survival rates were 91.8% for those diagnosed in 1991–5, 95.2% for those diagnosed in 1996–2000, and 95.4% for those diagnosed in 2001–6.

## DISCUSSION

The purpose of this study was to describe the time trends in the incidence, mortality, and 5-year relative survival of thyroid cancer in Israel during the period 1980–2012, in light of the global trends. Israel Cancer Registry data indicated a significant increase in the incidence of thyroid cancer in the study period overall (APCs 3.98–6.93), which was driven almost entirely by papillary cancer (APCs 5.75–8.86), while rates of other types of thyroid cancer remained stable or even decreased. Furthermore, higher rates of early detection were noted; the proportion of cases with local involvement at diagnosis almost doubled (from 28.8% to 56.1%) in the period 2000 through 2012, while the proportion of metastatic cases at diagnosis dropped considerably (from 10.9% in 2000 to 1.7% in 2012).

Thyroid cancer is a relatively rare cause of cancer death. During the study period we observed a modest reduction in thyroid cancer mortality in Jewish women and no substantial change in Jewish men. The small number of thyroid cancer deaths in the Arab population (0–5 annually) precluded trend analysis. Five-year relative survival after thyroid cancer diagnosis has modestly increased and is currently 90% and over in both population groups and both genders.

### Thyroid Cancer Incidence

The increasing trends of thyroid cancer incidence in Israel, for both genders and both population groups, closely follow the global trends. The statistically significant difference in age at diagnosis observed in Israel between Jews and Arabs is most likely due to the different age distribution of these population groups, with the Arab population being much younger.^16^

In 2015 La Vecchia et al.^10^ described thyroid cancer mortality and incidence in 28 European countries and in 20 countries, including Israel, from other parts of the world, based on information derived from the World Health Organization online database, for the period 1970–2012. Their conclusion was that the continuous rise in the incidence of thyroid cancer over the last few decades is evident in various countries worldwide.^10^ Indeed, in a recent publication referring to the global increase in thyroid cancer,^17^ annual percent changes in the incidence of thyroid cancer in Australia were 4.0 (1982–2007) for men, and 2.0 (1982–2000) and 13.8 (2000–7) for women. In Canada, for the time period 1970–96, the corresponding rates were 3.2 and 3.6, and for the time period 2002–8, 8.4 and 7.3, for men and for women, respectively. In China, APC for men was 2.6 (1983–2000) and for women, 4.9 (1983–2003). In the United Kingdom, APC 1998–2005) were 0.6 for men and 2.3 for women. The rates in the US for the period 1993–2008 were 6.3 and 7.0, for men and women, respectively.^17^ The Israeli figures are comparable (APCs for the period 1980–2012 were 4.0, 4.2, and 6.9 for Jewish men, Jewish women, and Arab women, respectively).

The fact that the main increase in thyroid cancer globally is derived from a rise in papillary carcinoma, the most common type of thyroid cancer, is also well established.^18^–^22^ The current Israeli status reflects this trend as well.

Thyroid cancer is associated with ionizing radiation, especially during childhood. This exposure may produce genetic instability and trigger genetic alterations involving the activation or inactivation of certain genes which had been associated with thyroid tumors: BRAF mutation and RET gene rearrangements in papillary carcinomas, RAS and PPARg-PAX8 mutations in follicular tumors, and p53 mutations in poorly differentiated and anaplastic carcinoma.^23^ Iodine intake influences the distribution of thyroid cancer by histological type rather than directly affecting the overall incidence, with more follicular and fewer papillary carcinomas in iodine-deficient areas.^17^ Hashimoto thyroiditis, on the other hand, is associated with higher risk for papillary carcinoma of the thyroid.^17^ Environmental exposures have also been suggested as risk factors for thyroid cancer, with volcanic environment associated with increased risk for papillary carcinoma.^17^ However, the reasons for the global increase in thyroid cancer incidence, and in the incidence of papillary carcinoma in particular, are not fully understood.

The increase may be a true one, due to a higher and/or a more frequent exposure to known and/or yet unknown risk factors for the disease. An alternative explanation is that the apparent increase in thyroid cancer incidence is artificial, the result of a change in diagnostic criteria, improved diagnostic techniques, and more aggressive follow-up of incidental findings, exposing a reservoir of indolent, subclinical tumors. As is the case with breast cancer and prostate cancer, over-diagnosis of thyroid cancer, that is, the diagnosis of small, clinically non-relevant tumors that would have otherwise remained asymptomatic,^24^ is a real concern. If the observed rise in thyroid cancer rates were due entirely to a genuine increase in thyroid cancer incidence, one would expect that the increase would be across most histological types, with no substantial change in the distribution of the disease staging at diagnosis, and accompanied by an increase in thyroid cancer mortality rates, as a result of an increased number of incident and prevalent cases in the population. If, however, the observed increases in thyroid cancer incidence are due in part to over-diagnosis, one would expect increases in specific histological types (slow-growing and less aggressive), as well as in the diagnosis of smaller, earlier-stage tumors, and steady if not decreased mortality rates as a result of earlier detection and over-diagnosis.

The debate is still open;^17^,^25^ some studies strongly support the hypothesis of over-diagnosis and incidental findings as the main explanation of the increased incidence of thyroid cancer overall, and of papillary carcinoma in particular.^26^–^29^ Others adopt a more conservative approach and suggest that the observed trends represent a genuine increase in thyroid cancer incidence.^30^–^32^ The Israeli findings may reflect both possibilities.

Utilization of advanced medical imaging techniques has increased in the last decades in Israel^33^ and other Westernized countries, e.g. the US.^34^ Increased usage of medical imaging may lead to incidental diagnosis of thyroid nodules, for example in the course of carotid artery imaging for the management of vascular disease. The increase in the incidence of early-stage tumors in Israel and the essentially stable mortality support the existence of incidental findings and over-diagnosis.

On the other hand, higher exposures to well-established and potential risk factors, specifically increased exposure to diagnostic and therapeutic radiation, are also likely explanations of the increasing incidence of thyroid cancer in the world and in Israel, and imply a genuine risk change. The significant increases over time in the use of medical diagnostic radiation, especially CT scans, in the world (and in Israel^33^) have been seen not only in adults but among children as well.^35^ Since the young thyroid gland is very sensitive to radiation,^36^ this may create a subpopulation susceptible to thyroid cancer in the long run. Furthermore, therapeutic radiation is a common modality in the treatment of certain cancers, childhood cancers included. The high survival rates of Israeli cancer patients in general, and of childhood cancer patients in particular,^37^ put the survivors at a higher risk for thyroid cancer later on.

Other risk factors suspected in the rise in thyroid cancer incidence include increases in body mass index and height.^5^–^7^ Obesity rates in Israel have grown in the last decades^38^ and are currently high and comparable to those seen in other Westernized countries.^39^ Height has been shown to increase with time in different ethnic groups in Israel. Furthermore, the results suggested that growth rates have been found to be higher following immigration to Israel, such that, among ethnic subgroups, growth rates in those born in Israel are higher compared to the rates in those immigrating to Israel.^40^

Additionally, several exposures may be unique to Israel and partially explain the high rates observed, relative to other countries in the world. For example, ionizing radiation, now a well-known and established risk factor for thyroid cancer, was regarded in the first half of the twentieth century as the state-of-the-art treatment for several benign conditions; follow-up of some patient cohorts receiving such treatments has indicated a relationship between radiation therapy and thyroid cancer.^41^ In Israel, many children who arrived in the massive immigration wave that occurred in the first decade following the establishment of the State (1948–58) received radiation for the treatment of tinea capitis and other conditions. The treated population was later found to be at a higher risk for thyroid^42^,^43^ and other cancers^44^ compared to the general population. This may have led to a cohort effect in the incidence of thyroid cancer in Israel. In 2012, the highest age-specific rates of thyroid cancer in Jewish men and women occurred in the expected age range of those who immigrated to the young State of Israel as children.

Use of fertility drugs^45^ and changes in reproductive patterns^7^,^8^ have also been suggested as potential risk factors for thyroid cancer. Fertility treatments in Israel are fully covered by the National Health Insurance Law and are provided universally up to the second child and/or maternal age of 51 years (with ovum donation for women 45–51 years of age). For this reason, Israel ranks highest in the world with respect to *in vitro* fertilization (IVF) treatments per capita. However, success rates are in fact lower than in Europe and the US, probably because many IVF treatments are delivered to patients who are legally entitled to receive treatment but clinically have low potential for success.^46^ Thus, women in Israel are more likely than women elsewhere to receive fertility treatments, and to repeat them. If thyroid cancer is indeed associated with fertility treatments, this may add to the susceptibility of Israeli women to the disease.

### Thyroid Cancer Mortality

La Vecchia et al.,^10^ in their study on global mortality and incidence trends of thyroid cancer in 1970 through 2012, concluded that thyroid cancer death rates vary worldwide, the highest rates occurring in Central America and Asia (Israel included) as well as Central and Eastern Europe, and the lowest rates being observed in Western Europe and North America. Additionally, they noted that most countries that historically presented with high rates of thyroid cancer mortality have experienced a reduction in these rates; however, countries that historically presented with the lowest rates of thyroid cancer mortality (mostly in North America and Northern Europe) have seen a plateau in mortality over the last two decades.^10^ Our findings indicate a stable trend in thyroid cancer mortality in Jewish men and a modest but significant reduction in Jewish women during the study period. Similar trends have also been reported for England;^47^ thus the Israeli picture is in accordance with the reported global trends.^10^

Overall, mortality rates attributed to thyroid cancer in Israel are low (ASR 0.4–0.7/100,000); nevertheless Israel is ranked among the 10 countries in the world with the highest rates of thyroid cancer mortality. One of the reasons may be the fact that mortality rates, computed as the number of deaths attributed to a certain condition divided by the total population at risk, reflect to a certain extent the incidence of this condition in this specific population; indeed, Israel is ranked amongst the top 10 countries in the world also with respect to thyroid cancer incidence. In addition, although the proportion of localized tumors at diagnosis doubled in Israel between 2000 and 2012, it amounted to 56% of all cases; in the US the proportion of localized thyroid tumors in 1991 was 61%,^2^ and 68% in 2005–11.^48^ A higher proportion of more advanced tumors at diagnosis may also impact mortality rates.

The observed difference of thyroid cancer mortality rates by gender in Israel may reflect the fact that the increase in incidence trends is higher in women compared to men^17^,^47^ and thus may represent, at least partially, a higher proportion of over-diagnosis in women, which is expected to result in decreasing mortality rates.^49^ The differences in the mean age of death between Jews and Arabs may be explained by the different mean ages of diagnosis in the two population groups, mostly reflecting their different age distribution.^16^

### Thyroid Cancer 5-Year Relative Survival

In light of the increasing incidence and stable or decreasing mortality trends of thyroid cancer in Israel, an increase in the survival is expected. Indeed, we observed a modest increase in the relative 5-year survival of thyroid cancer in Israel, from 84%–92% in subjects diagnosed in 1991–5 to 95%– 96% in subjects diagnosed in 2001–6. Similar trends, albeit with higher percentages, were reported from the US, where the relative 5-year survival for subjects diagnosed in 1989–94 was 95% and for subjects diagnosed in 2005–11, 98%.^2^

In conclusion, the Israeli picture with respect to thyroid cancer incidence and mortality closely follows global trends. Based on the Israeli findings, it appears that the observed increase in incidence is derived from both incidental findings and over-diagnosis and from true increase in disease incidence. The trends should be monitored closely, and these results call for an analytical study to assess potential risk and prognostic factors relevant to Israeli subjects.

## Abbreviations:

APC: annual percent change
CBS: Central Bureau of Statistics
INCR: Israel National Cancer Institute
IVF: *in-vitro* fertilization
SEER: Surveillance, Epidemiology and End-results.
